# Metastatic Liposarcoma: A Cause of Symptomatic Acute Pericarditis

**DOI:** 10.1080/13577149778281

**Published:** 1997-12

**Authors:** Matthew Q. F. Hatton, Robin Reid, Ann Barrett

**Affiliations:** 1 Beatson Oncology Centre Western Infirmary Dumbarton Road Glasgow G11 6NT UK; 2 Department of Pathology Western Infirmary Glasgow UK

## Abstract

We describe a patient presenting with a myxoid liposarcoma of the lower thigh in whom an episode of acute pericarditis indicated the recurrence of widespread metastatic disease.


*Sarcoma* (1997) 1, 181–182

CASE REPORT

### Metastatic liposarcoma: a cause of symptomatic acute pericarditis

MATTHEW Q. F. HATTON,[1] ROBIN REID[2] & ANN BARRETT[1]

[1]*Beatson Oncology Centre,* [2]*Department of Pathology, Western Infirmary, Glasgow, UK*

**Abstract**
We describe a patient presenting with a myxoid liposarcoma of the lower thigh in whom an episode of acute pericarditis indicated the recurrence of widespread metastatic disease.

**Key words:** *myxoid liposarcoma, knee, recurrent metastases, acute pericarditis.*

### Introduction

Cardiac metastases are unusual and most commonly of lung or breast origin with soft tissue sarcomas an occasionally cited cause. Pericardial involvement occurred in two-thirds of these cases with isolated pericardial disease accounting for 45% of cardiac metastases. In most cases, pericardial involvement is asymptomatic[1] and documented at post-mortem.[2] Symptomatic presentations vary from acute cardiac tamponade to a syndrome similar to cancer cachexia. Although a wide range of other symptoms has been reported, chest pain is rarely a prominent feature.[3]

### Patient

A 39-year-old man presented with a 5-year history of swelling on the postero-medial aspect of the left knee. A 7-cm soft swelling arising from the lower end of vastus medialis was found on examination. The tumour was removed by a marginal resection and histology showed a myxoid liposarcoma with small foci of round cell transformation, of Trojani grade 2.[4]

Thirty-three months later, the patient had a histologically confirmed relapse in the left upper deep cervical region and whole-body computed tomography (CT) showed a mass in the right iliac fossa. There was no evidence of local recurrence. Intravenous chemotherapy (adriamycin, ifosphamide) led to a complete regression of the neck swelling and a partial response of the right iliac fossa mass. Macroscopic removal of the residual abdominal tumour was achieved at subsequent laparotomy. Isolated relapses at both these sites occurred over the next 18 months. The neck was treated with radiotherapy (50 Gy in 25 fractions) following excision biopsy and a further resection was performed for the abdominal recurrence.

Five months after this surgery, hospital admission was precipitated by severe, central chest pain, eased by sitting forward associated with nausea, vomiting and 'flu-like' symptoms. The electrocardiogram showed widespread S–T wave elevation consistent with acute pericarditis. A pericardial effusion was the only abnormality seen on echocardiography. Symptoms settled quickly and (following review by a cardiologist) the patient was discharged with a provisional diagnosis of viral pericarditis. However, this was not confirmed serologically and a CT scan of the chest subsequently showed widespread recurrence of the liposarcoma with thickening of the pericardial wall, a moderate pericardial effusion, intra-abdominal and pelvic disease, and small lung metastases. A trial of oral etoposide chemotherapy was unsuccessful, although with symptomatic treatment a good quality of life was maintained, with no further cardiac problems, until the patient died a year later, 7 years after his original presentation.

### Discussion

Soft tissue sarcomas are malignant neoplasms arising from connective tissue, accounting for less than 1% of all malignancies. Liposarcomas are one of the more common forms of soft tissue sarcoma, accounting for 10–16%.[5] The management of sarcomas tends to be based on site, grade and stage, rather than histology, although it is important to

Correspondence to: M. Hatton, Beatson Oncology Centre, Western Infirmary, Dumbarton Road, Glasgow G11 6NT, UK. Tel: +44 (0)141 211 2000; Fax: +44 (0)141 337 1712.

1357-714X/97/030181-02 $9.00 © 1997 Carfax Publishing Ltd note that some variations exist between the different histological groups. One variation described is the tendency for the initial metastases in liposarcomas to occur at extrapulmonary sites in up to 59% of patients,[6] with myxoid liposarcoma, in particular, producing secondary lesions on serosal surfaces, including the pericardium.

Malignant sarcomas rarely involve the heart or pericardium. Approximately 0.1% have a primary site in the heart or mediastinum, of which liposarcomas account for 12.5%.[7] Reports of isolated metastatic liposarcoma involving the heart are, perhaps, confined to eight cases,[6,8] and, surprisingly, pericardial involvement in the context of a widespread relapse seems less well documented.

Our case is made more noteworthy by its presentation with the severe chest pain of acute pericarditis and the transient symptoms. The incidence of symptomatic malignant cardiac involvement is difficult to estimate as there is a wide difference in the reported rates in clinical[9] and pathological[2] series, suggesting that a large number of patients with cardiac metastases are asymptomatic. When symptoms do occur, they tend to be those associated with cardiac tamponade, in particular dyspnoea, with chest pain reported in only 18% of patients.[3]

Non-malignant causes of pericardial disease are common in cancer patients.[2] Changes on electrocardiography are not specific and echocardiography is better able to differentiate malignant from non-malignant causes. In our case, it was the findings of the CT that spared the more invasive investigations of pericardial aspiration with cytological examination.

The prognosis for cardiac malignant involvement is very poor, with a median survival of 3 months.[3] Treatment recommendations include the surgical removal of an isolated cardiac metastasis, radiation, combination chemotherapy, pericardiodesis and catheter drainage.[2] The cardiac symptoms experienced by our patient settled spontaneously, and treatment was directed at maintaining his good quality of life. He experienced no further cardiac symptoms and required no specific cardiac intervention before the abdominal disease progressed to cause death a year after his episode of acute pericarditis.

### References

1 Buck M, Ingle JN, Giuliani ER, *et al.* Pericardial effusion in women with breast cancer. *Cancer* 1987; 60:263–9.
2 Hagemeister FB, Buzdar AU, Luna MA, *et al.* Causes of death in breast cancer. *Cancer* 1980; 46:162–7.
3 Reitknecht F, Regal AM, Antkowiak JG, *et al.* Management of cardiac tamponade in patients with malignancy. *J Surg Oncol* 1985; 30:19–22.
4 Trajani M, Contessa G, Coindre JM, *et al.* Soft tissue sarcoma of adult: study of pathological and prognostic variables and definition of a histological grading system. *Int J Cancer* 1984; 33:37–41.
5 Enzinger FM, Weiss SW, eds. *Soft tissue tumours.* 3rd edn. St Louis: Mosby, 1994:431–66.
6 Cheng EY, Springfield DS, Mankin HJ. Frequent incidence of extrapulmonary sites of initial metastasis in patients with liposarcoma. *Cancer* 1995; 75:1120–7.
7 Mack TM. Sarcomas and other malignancies of soft tissue, retroperitoneum, peritoneum, pleura, heart, mediastinum and spleen. *Cancer* 1995; 75 (suppl.):220–44.
8 Papa MZ, Shinfeld A, Klein E, *et al.* Cardiac metastasis of liposarcoma. *J Surg Oncol* 1994; 55:132–4.
9 Bitran JD, Evans R, Brown C. The management of cardiac tamponade in patients with breast cancer. *J Surg Oncol* 1984; 27:42–4.
